# Deep Learning Feature Extraction Approach for Hematopoietic Cancer Subtype Classification

**DOI:** 10.3390/ijerph18042197

**Published:** 2021-02-23

**Authors:** Kwang Ho Park, Erdenebileg Batbaatar, Yongjun Piao, Nipon Theera-Umpon, Keun Ho Ryu

**Affiliations:** 1 Database and Bioinformatics Laboratory, College of Electrical and Computer Engineering, Chungbuk National University, Cheongju 28644, Korea; khblack@dblab.chungbuk.ac.kr (K.H.P.); eegii@dblab.chungbuk.ac.kr (E.B.); 2 School of Medicine, Nankai University, Tianjin 300071, China; ypiao@nankai.edu.cn; 3 Department of Electrical Engineering, Faculty of Engineering, Chiang Mai University, Chiang Mai 50200, Thailand; 4 Biomedical Engineering Institute, Chiang Mai University, Chiang Mai 50200, Thailand; 5 Data Science Laboratory, Faculty of Information Technology, Ton Duc Thang University, Ho Chi Minh 700000, Vietnam; 6 Department of Computer Science, College of Electrical and Computer Engineering, Chungbuk National University, Cheongju 28644, Korea

**Keywords:** hematopoietic cancer, cancer classification, subtype classification, machine learning, autoencoder, data mining, bioinformatics

## Abstract

Hematopoietic cancer is a malignant transformation in immune system cells. Hematopoietic cancer is characterized by the cells that are expressed, so it is usually difficult to distinguish its heterogeneities in the hematopoiesis process. Traditional approaches for cancer subtyping use statistical techniques. Furthermore, due to the overfitting problem of small samples, in case of a minor cancer, it does not have enough sample material for building a classification model. Therefore, we propose not only to build a classification model for five major subtypes using two kinds of losses, namely reconstruction loss and classification loss, but also to extract suitable features using a deep autoencoder. Furthermore, for considering the data imbalance problem, we apply an oversampling algorithm, the synthetic minority oversampling technique (SMOTE). For validation of our proposed autoencoder-based feature extraction approach for hematopoietic cancer subtype classification, we compared other traditional feature selection algorithms (principal component analysis, non-negative matrix factorization) and classification algorithms with the SMOTE oversampling approach. Additionally, we used the Shapley Additive exPlanations (SHAP) interpretation technique in our model to explain the important gene/protein for hematopoietic cancer subtype classification. Furthermore, we compared five widely used classification algorithms, including logistic regression, random forest, k-nearest neighbor, artificial neural network and support vector machine. The results of autoencoder-based feature extraction approaches showed good performance, and the best result was the SMOTE oversampling-applied support vector machine algorithm consider both focal loss and reconstruction loss as the loss function for autoencoder (AE) feature selection approach, which produced 97.01% accuracy, 92.60% recall, 99.52% specificity, 93.54% F1-measure, 97.87% G-mean and 95.46% index of balanced accuracy as subtype classification performance measures.

## 1. Introduction

Lots of bioinformatics techniques have been developed for the disease detection and diagnosis of patients with incurable diseases such as cancer for several decades [1]. However, it still remains challenging to deal with cancer patients. Furthermore, the development of appropriate classification models using the gene which is extracted from patients is useful for early diagnosis of both patients and normal people. Cancer is a major disease which causes death, involving abnormal cell differentiation [2]. It is caused by many reasons, but the majority of cancers (90~95%) are due to genetic mutations from lifestyle factors such as smoking, obesity, alcohol and so on. The remaining 5~10% are caused due to inherited genes [3].

A hematopoietic malignancy is a neoplasm from hematopoietic cells in the bone marrow, lymph nodes, peripheral blood and lymphatic system which is related to organs of the hematopoietic system. Furthermore, it can be found in other organs such as the gastrointestinal system and the central nervous system [4]. Given that hematopoietic malignancies occur in the hematopoietic system, this malignancy is called a liquid tumor. While uncommon in other cancers, chromosomal translocations are a common cause of these diseases. Hematopoietic cancer accounts for 8~10% of all cancer diagnoses, and its mortality rate is also similar to this [5].

Historically, hematopoietic cancer is divided by whether the malignant location is in the blood or the lymph nodes. However, in 2001, the World Health Organization (WHO) introduced the WHO classification of tumors of hematopoietic and lymphoid tissue as a standard and updated it in 2008 and 2016 [6]. This WHO classification criterion focused on cell linkage rather than the location of the occurrence. According to the WHO classification, hematopoietic malignancies are mainly divided into leukemia, lymphoma and myeloma [7].

Leukemia is one type of hematopoietic cancer that results from genetic changes in hematopoietic cells in the blood or bone marrow. If an abnormality occurs in the bone marrow, the abnormally generated blood cells mix with blood in the body and spread widely into the body through the blood stream [8]. Most leukemia cases are diagnosed in adults aged over 65 years, but it is also commonly observed in children under the age of 15 [7]. The American Cancer Society (ACS) reported, in 2020, that the United States will see about 60,530 new cases and 23,100 deaths from leukemia [9]. Lymphoma is usually found in distinct stationary masses of lymphocytes, such as the lymph node, thymus or spleen. Like leukemia, lymphoma can also travel through the whole body by the blood stream. Commonly, lymphoma cases are divided into Hodgkin lymphoma, non-Hodgkin lymphoma, acquired immune deficiency syndrome (AIDS)-related lymphoma and primary central nervous system (CNS) lymphoma [8]. In 2020, the ACS reported that there will be approximately 85,720 new cases and 20,910 deaths from lymphoma [9]. Myeloma is a tumor that occurs in plasma cells which are differentiated from bone marrow, blood or other tissue. Plasma cells generate antibodies that protect against disease or infection, but when they develop abnormally, it interferes with antibody generation and causes confusion in the human immune system [8]. According to the estimation by the ACS, there would be 32,270 new cases and 12,830 deaths in 2020 [9].

There have been several studies conducted using genetic data-based cancer classification by machine learning and data mining approaches [10,11,12,13,14,15,16,17,18]. For instance, one study [10] utilized several machine learning approaches with naïve Bayes and k-nearest neighbor classification algorithm for breast cancer classification. Another study [11] applied classification algorithms for binary subtype cancer classification on acute myeloblastic leukemia microarray data. Other kinds of research also typically use machine learning algorithms such as support vector machine, decision tree, random forest, lasso and neural network [12,13,14,15,16,17,18].

Over the years, various approaches for data mining have been applied on many cancer research studies. Specifically, a deep learning method was applied in this area [19,20,21,22,23]. Ahmed M et al. [19] developed a breast cancer classification model using deep belief networks in an unsupervised part for learning input feature statistics. Additionally, in the supervised part, they adopted a conjugate gradient and Levenberg–Marquardt algorithm.

Furthermore, there are several studies on cancer subtype classification. A study [20] used a deep learning approach for kidney cancer subtype classification using miRNA data from The Cancer Genome Atlas (TCGA), which contained five different subtypes. They employed neighborhood component analysis for feature extraction and a long short-term memory (LSTM)-based classifier. DeepCC [22] architecture using the gene set enrichment analysis (GSEA) method and artificial neural network generated deep cancer subtype classification frameworks that made comparisons between machine learning algorithms such as support vector machine, logistic regression and gradient boost using a colon cancer dataset from TCGA which contains 14 subtype cancer labels, and a Deeptype [23] framework has been made for cancer subtype classification based on PAM50 [24]. They used a multi-layer neural network structure for adopting representation power to project on representation space.

Traditional approaches [10,11,12,13,14,15,16,17,18] have used statistical techniques for cancer classification. Sun et al. [17] used an entropy-based approach for feature extraction in a cancer classification model. However, this method has a disadvantage; that is, multiple classes cannot be applied at once because each cancer is classified one by one through binary classification for cancer classification. In the case of Deeptype [23], clustering is established using a specific gene set called PAM50, previously known for breast cancer.

However, since this PAM50 indicator is used as an already known indicator, subtypes can be classified through some valid information about breast cancer. Above all, for other cancers, including hematopoietic cancer, there is no gene set for subtype classification. In view of this, our work has a difference in feature extraction and subtype classification only from the gene expression data of hematologic cancer. In order to overcome the demerit of the multi-class classification task and the limitations due to the absence of a gene set, we applied a method of feature extraction using an autoencoder-based method among deep learning methods. In addition, we propose a subtype classification method in which reconstruction error is generated by the autoencoder. The classification error generated by the classification model and merged error are used as the loss function by referring to the loss function application methods of Deeptype [23].

The goal of this work is to develop an autoencoder-based feature extraction approach for hematopoietic cancer subtype classification. We not only focus on the five subtypes of hematopoietic cancer and conduct a study on classifying by applying deep learning techniques, but we also perform a feature extraction and calculation of two kinds of errors, which are reconstruction error and classification error. In the process, first a deep autoencoder approach is used for extracting suited features for building a classification model, and then the reconstruction error and classification error (cross-entropy loss and focal loss) are calculated for considering the data imbalance problem when building the classification model using the extracted features. To validate the deep autoencoder-based classification model, we compared other traditional feature selection algorithms and classification algorithms with an oversampling approach. We compared five widely used classification algorithms including logistic regression (LR), random forest (RF), k-nearest neighbor (KNN), artificial neural network (ANN) and support vector machine (SVM).

We compared our proposed method with traditional cancer classification and cancer subtype classification methods such as data mining and machine learning approaches which are not able to be used in the previous end-to-end approaches. Our end-to-end approach has multiple steps including feature engineering, data imbalance handling and a classification task. The objectives of this study are to extract features from a deep learning-based approach on the gene expression data for predicting hematopoietic cancer subtypes and develop an end-to-end deep learning-based classification model. The major contributions of this study are, briefly, as follows:

- We propose an end-to-end approach without any manual engineering, which classifies hematopoietic cancer subtypes;
- We adopt a non-linear transformation step by using a deep autoencoder to select deep features from gene expression data in hematopoietic cancer by adopting a deep learning architecture-based feature engineering task;
- We implement a mixed loss function for the proposed deep learning model, considering both the compression of knowledge representation and the data imbalance problem.

The remainder of this paper is organized as follows: Section 2 introduces the hematopoietic cancer gene expression dataset from TCGA. Furthermore, the proposed deep autoencoder-based approach is explained in detail. In Section 3, the experimental results are provided. Finally, Section 4 discusses the experimental results with our conclusion.

## 2. Materials and Methods

### 2.1. Dataset

TCGA is a site that contains plenty of information and data related to human cancer. Currently, as of 2020, there are 47 types of cancer data, and each cancer’s data are provided various kinds of data such as gene expression, clinical data and methylation data from large numbers of patient with cancer [25]. Although some raw data, which include original sequence information, are treated by controlled data that have to be approved for use in experiments by TCGA, most data are freely accessible for researchers. We collected TCGA data from 2457 patients with hematopoietic cancer gene expression data. The collected gene expression data have five subtypes of hematopoietic cancer: lymphoid leukemia, myeloid leukemia, leukemia not otherwise specified (nos), mature B-cell leukemia and plasma cell neoplasm. The size of each hematopoietic subtype sample is 550 lymphoid leukemia cases, 818 myeloid leukemia, 104 leukemia nos, 113 mature B-cell leukemia and 860 plasma cell neoplasm. Furthermore, these data have 60,453 exons’ information with one gene expression profiling measurement. The level of gene expression is fragments per kilobase per million (FPKM) mapped measure [26]. This FPKM can be calculated by the following equation:

(1) $$FPKM=\frac{Total\;fragments\;of\;interest}{Mapped\;reads\;\left(Millions\right)\times exon\;length\;\left(kb\right)}$$

FPKM is a normalized estimation of gene expression based on RNA-seq data considering both the number of reads and the length of the exon, measured by kilobase unit. That is, a large FPKM means a large amount of expression per unit length, so the FPKM of a certain gene refers to a relative amount of gene expression.

The statistics of hematopoietic cancer are shown in Table 1. In a preprocessing step, we eliminated noisy and non-valued instances. These preprocessed data were used for the subtype classification in this experiment; they were divided into 80% for training and 20% for testing. However, as we introduced above, the dataset was considerably imbalanced.

Due to this data imbalance problem, we applied a cost function on the classification and feature extraction and oversampling method. We also used an autoencoder-based model for extracting the highly related gene expression data and compared this algorithm with other traditional dimension reduction algorithms.

### 2.2. Proposed Autoencoder-Based Approach

**Figure 1:**
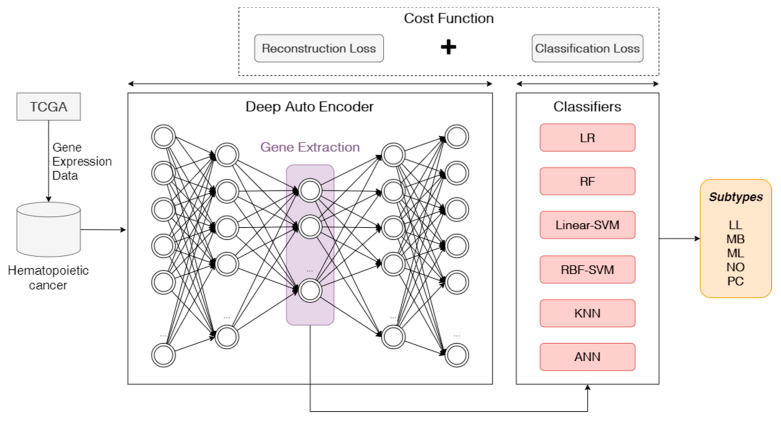
Overview of proposed autoencoder-based classification approach. We used hematopoietic cancer gene expression data from The Cancer Genome Atlas (TCGA). The deep autoencoder (DAE) model was used to extract deep features from these gene expression data as a lower-dimensional vector. In this study, we use an autoencoder (AE) and a variational autoencoder (VAE) as DAEs. The classifier is used to classify hematopoietic cancer subtypes. We summed the reconstruction loss on DAE and classification loss in the cost function. LR: logistic regression; RF: random forest; SVM: support vector machine; RBF: radial-based function; KNN: k-nearest neighbor; ANN: artificial neural network.

In the experiment, we propose a deep learning-based hematopoietic cancer subtype classification approach. Figure 1 shows the proposed approach which inputs the hematopoietic cancer gene expression data from TCGA and outputs the subtype classification result. This approach consists of an autoencoder feature extraction part and a machine learning-based cancer subtype classifier.

In the DAE structure, we employed the mean squared error (MSE) for measuring deep learning reconstruction loss when training the training set and adopted focal loss [27] as a measurement of the classification error in the classifier. Focal loss (FL) is an updated version of cross-entropy loss, which was used for class imbalance encountered during the model training. Therefore, our proposed autoencoder-based hematopoietic cancer subtype classification approach used the sum of both MSE as reconstruction loss and FL as classification loss as a cost function for this approach.

We performed this experiment on an Intel Xeon E3 1231 v3 processor with 32G memory and RTX 2060 (Gigabyte, New Taipei City, Taiwan). Additionally, we used Python 3.7 for parsing the data and analysis by implementing deep learning and machine learning libraries. The whole process of this experiment and the methodologies including the machine learning and deep learning approaches performed are explained in detail in the next section.

#### 2.2.1. Feature Extraction using Deep Learning Approach on Gene Expression Data

In this research, we used a DAE-based feature selection approach. The autoencoder structure has a strong point in the non-linear feature selection and transformation. Additionally, we compared this DAE-based approach with traditional statistical-based feature selection approaches, which are Principal Component Analysis (PCA) [28] and Non-negative Matrix Factorization (NMF) [29]. PCA is one of most popular statistical techniques which relates factor analysis with multivariate analysis. This algorithm aims to represent the characteristics in a dataset as a small set of factors or a dataset that keeps important information. Furthermore, NMF is available for multivariate analysis. This algorithm based on linear algebra makes complex feature information into smaller non-negative matrices. Generally, PCA tends to group both positively and negatively correlated components; on the other hand, NMF divides factors into positive vectors. These kinds of statistical factor analyses access the linearity constraint, so we applied DAE techniques with non-linear calculations for obtaining better classification results.

We applied two DAE models. One was a normal autoencoder model (AE) [30,31] and the other was a variational autoencoder model (VAE) [32,33]. Both of the two autoencoder models were constructed using the Pytorch deep learning library in Python [34]. The structure of an AE is divided into two main parts: encoder and decoder. The encoder has an input layer with three fully connected hidden layers with 2000, 500 and 100 nodes. The decoder is comprised of two hidden layers, which are fully connected. The details of the autoencoder are explained in Appendix A.

Furthermore, similar with the AE structure, VAEs also have an encoder and a decoder. The VAE is normally used in semi-supervised learning models nowadays. Additionally, it can learn approximate inferences and be trained using the gradient descent method. The main approach of the VAE is using probability distribution to obtain samples which match the distribution. The details of the variational autoencoder are explained in Appendix B.

#### 2.2.2. Hematopoietic Cancer Subtype Classification Model

For the subtype classification on hematopoietic cancer, we generated a feed-forward neural network (NN) which has one input layer, one hidden layer with 100 nodes and one output layer. The features we extracted from the DAE utilized the input of the NN. In here, we adopt ReLU and sigmoid for non-linear activation function. This NN has the two loss functions for measuring classification error, which is the error between the real value and the predicted value. The details of the NN are shown in Appendix C.

The two loss functions are cross-entropy loss (CE) and focal loss (FL). CE is the most widely used loss function in classification models. However, if there are plenty of class labels with the imbalance status, it incurs a loss with non-trivial magnitude. If there are a lot of easy examples to be classified, which means there is a large class imbalance on the dataset, the CE of major class labels is going to be larger than the minor classes.

For handling this class imbalance problem, a modulating factor is added to CE, defined as focal loss (FL). This modulating factor in focal loss is ${\left(1-p_{i}\right)}^{\gamma }$. Here, γ is a focusing factor which can be a changeable parameter that ranges γ≥0. Due to this advantage, using FL can prevent the overfitting problem which can accompanied by class imbalance. The details of CE and FL are described in Appendix D.

#### 2.2.3. Training the Models

The loss functions on autoencoder models are calculated by using the difference between the input and the output. The formulas of each loss function are shown below:

(2) $$L_{DAE}\left(input,\;reconstruction_{input}\right)=Mean\;Squared\;Error\;L_{NN}\left(hidden_{encode_{3}},\;output\right)=\{\begin{array}{c} Cross\;Entropy\;loss\\ Focal\;loss \end{array}\;L\left(input,\;output\right)=L_{DAE}+L_{NN}$$

In this experiment, we adopted the Adam optimizer [35] for updating the weight and bias iterative based in the training data. This approach has some merits. One is that the step size does not affect the gradient rescaling. Another is that Adam can refer to previous gradients for updating the step size. We performed several trials for defining the learning rate and set it to 0.0001. The batch size of the training set was 128, with the maximum epoch size set to 3000 with an early stopping approach for checking the optimal epoch number.

## 3. Experimental Results

### 3.1. Results of Feature Selection

We extracted key features on hematopoietic cancer gene expression data by using DAE approaches. To compare this result with traditional feature selection algorithms, we used PCA and NMF methods. For this comparison, we coded PCA and NMF algorithms using a Scikit-learn [36] and DAE model using a Python deep learning library, Pytorch [34].

**Figure 2:**
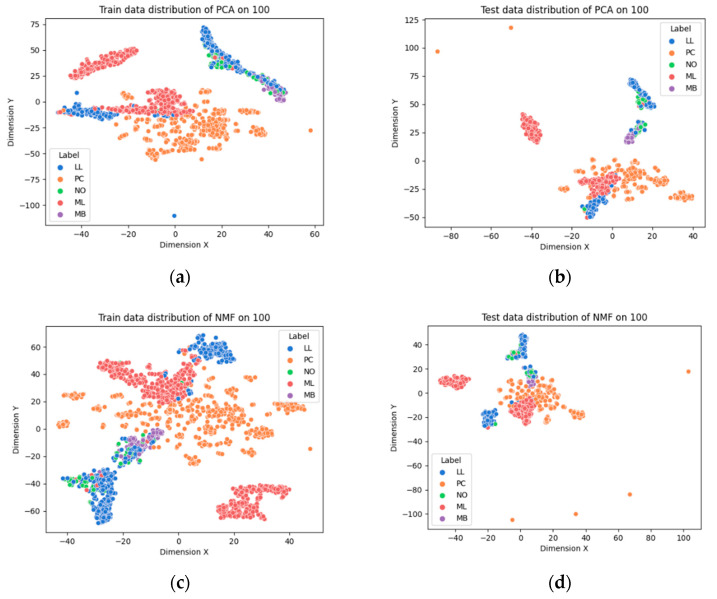

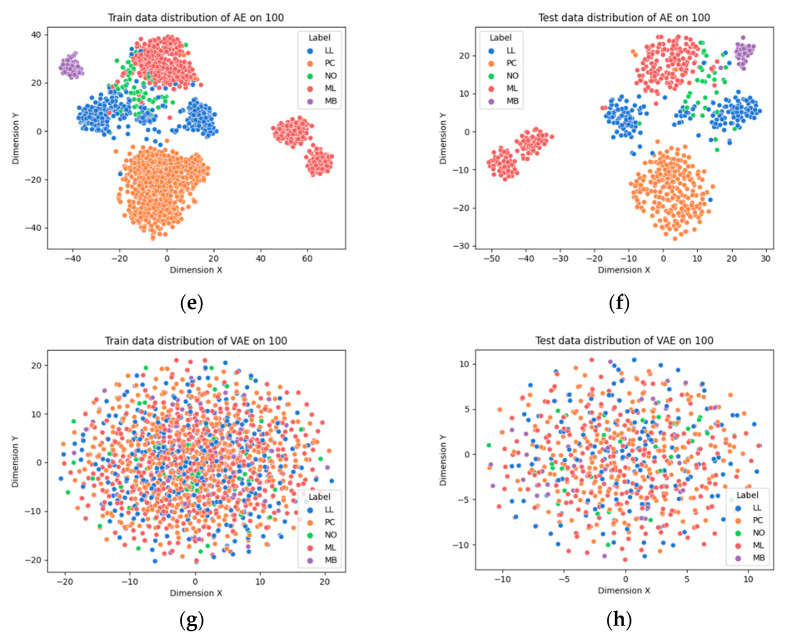
The extracted feature visualization from hematopoietic cancer gene expression data. (**a**,**b**) Visualization of training and test data extracted from principal component analysis (PCA), respectively. (**c**,**d**) Visualization of training and test data extracted from non-negative matrix factorization (NMF), respectively. (**e**,**f**) Visualization of training and test data extracted from the autoencoder (AE) using t-distributed stochastic neighbor embedding (tSNE), respectively. (**g**,**h**) Visualization of training and test data extracted from variational autoencoder (VAE) using tSNE, respectively. LL: lymphoid leukemia; PC: plasma cell neoplasm; NO: leukemia not otherwise specified (nos); ML: myeloid leukemia; MB: mature B-cell leukemia.

For pair comparison, we used t-Distributed Stochastic Neighbor Embedding (tSNE) [37] which is used in the conversion of high-dimensional data visualization into low-dimensional embedding. It converts high-dimensional Euclidian distance between data points into conditional probabilities for mapping low-dimension space and adopts KL-divergence [38] to minimize mismatch on the low-dimensional data representation. Using this technique, researchers can acquire more interpretable data visualization on high-dimensional data. In this experiment, we used the tSNE technique for mapping data into a two-dimensional plane (dimension X, Y) for DAE feature selection, and the visualization of the extracted features of each approach is shown in Figure 2.

Among the visualizations of the selected gene expression data using several approaches, we can see that the result of the DAE-based AE model is the most clearly well distinguished compared with other results.

### 3.2. Results of DAE Training Process

**Figure 3:**
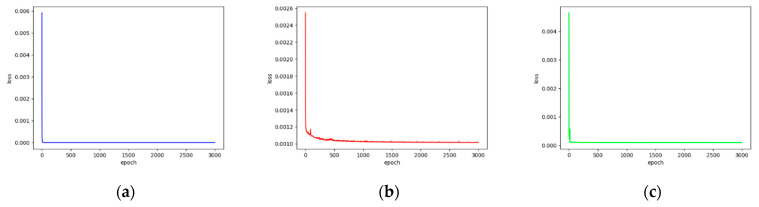
The training loss graphs on AE-based feature extraction. The mean squared error (MSE) was calculated as reconstruction loss and cross-entropy loss (CE) was calculated by classification loss. The x-axis represents the number of epochs and y-axis represents the loss value. (**a**) CE-based classification loss graph for each epoch (blue). The CE value is decreased until 5.32 × 10^−13^; (**b**) MSE-based reconstruction loss graph for each epoch (red). The MSE value is decreased until 0.0001. (**c**) Total loss (CE + MSE) graph for each epoch (green). The value of total loss is decreased until 0.0001.

**Figure 4:**
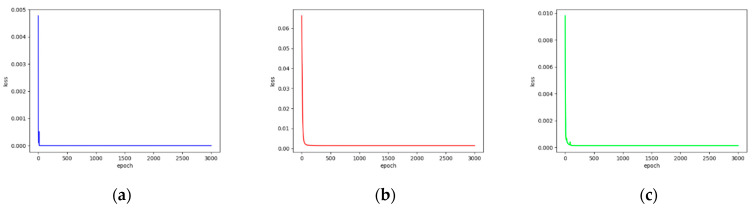
The training loss graphs on VAE-based feature extraction. The MSE was calculated as reconstruction loss, and CE was calculated by classification loss. The x-axis represents the number of epochs and y-axis represents the loss value. (**a**) CE-based classification loss graph for each epoch (blue). The CE value is decreased until 5.43 × 10^−13^. (**b**) MSE-based reconstruction loss graph for each epoch (red). The MSE value is decreased until 0.00013. (**c**) Total loss (CE + MSE) graph for each epoch (green). The value of total loss is decreased until 0.00014.

**Figure 5:**
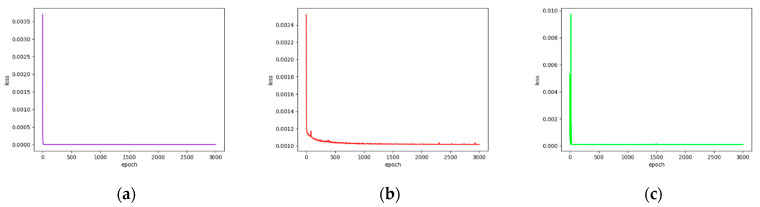
The training loss graphs on AE-based feature extraction. The MSE was calculated as reconstruction loss, and focal loss (FL) was calculated by classification loss. The x-axis represents the number of epochs and y-axis represents the loss value. (**a**) FL-based classification loss graph for each epoch (purple). The FL value is decreased until 5.97 × 10^−12^. (**b**) MSE-based reconstruction loss graph for each epoch (red). The MSE value is decreased until 0.001. (**c**) Total loss (FL + MSE) graph for each epoch (green). The value of total loss is decreased until 0.0001.

**Figure 6:**
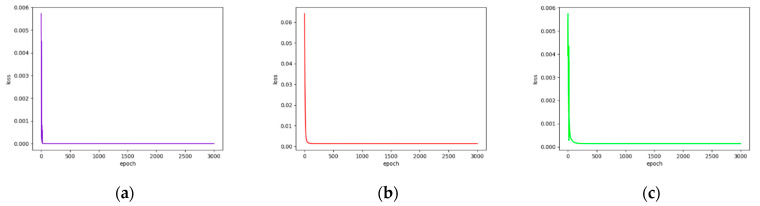
The training loss graphs on VAE-based feature extraction. The MSE was calculated as reconstruction loss, and FL was calculated by classification loss. The x-axis represents the number of epochs and y-axis represents the loss value. (**a**) FL-based classification loss graph for each epoch (purple). The FL value is decreased until 5.43 × 10^−13^; (**b**) MSE-based reconstruction loss graph for each epoch (red). The MSE value is decreased until 0.00014. (**c**) Total loss (FL + MSE) graph for each epoch (green). The value of total loss is decreased until 0.00014.

We extracted key features on hematopoietic cancer gene expression data by using DAE approaches which consist of AE and VAE. Each DAE approach was trained with 3000 epochs for each iteration. We also calculated the loss functions MSE, CE and FL. During the feature extraction process, we calculated the MSE from the AE model, and classification loss (CE and FL) was calculated on the model training class. Furthermore, the total loss was generated by merging MSE and classification loss. Figure 3 and Figure 4 show the loss function graphs for the cancer classification using MSE as the reconstruction error, CE and FL as the classification error and total error as the merged MSE and CE on AE and VAE, respectively. Figure 5 and Figure 6 show loss function graphs using MSE as the reconstruction error, FL as the classification error and total error as the calculated sum of MSE and FL on AE and VAE, respectively.

### 3.3. Hematopoietic Cancer Subtype Classification Model Interpretation

**Figure 7:**
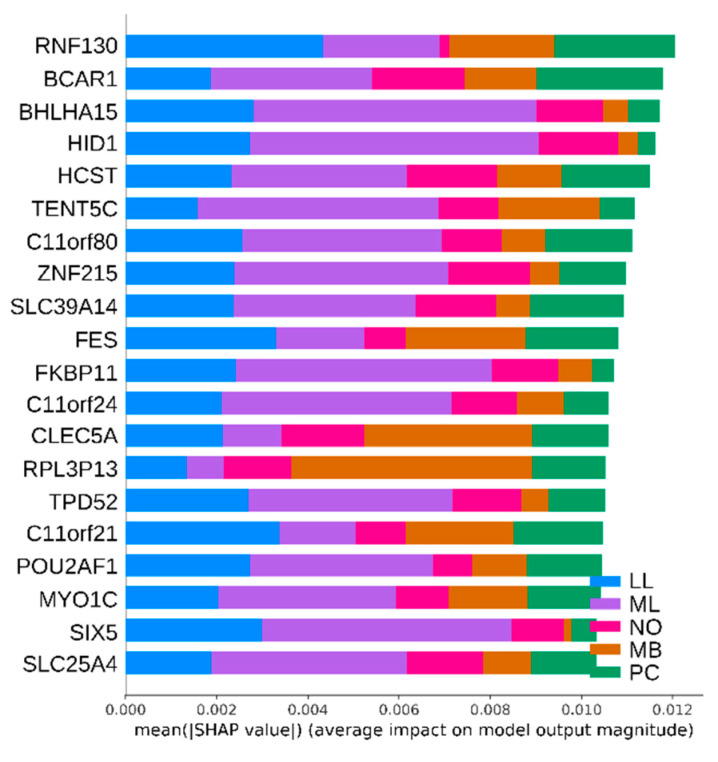
The SHAP summary plot for the top 20 important bio-markers for hematopoietic cancer subtype classification.

The Shapley Additive exPlanations (SHAP) method [39], which is based on game theory [40] and local explanations [41], is often used to describe a model’s output. The top 20 important bio-markers for hematopoietic cancer subtype classification, which were derived from the autoencoder, are shown in Table 2 and in Figure 7 as a SHAP summary plot.

As shown above in Figure 7, the most important bio-marker for hematopoietic cancer subtype classification is Ring Finger Protein 130 (RNF130). Including RNF130, the top 20 bio-markers are well known as oncogenes and some bio-markers are directly related to hematopoietic processes. For example, RNF130 is usually related to pathways on the innate immune system and Class I MHC (Major Histocompatibility Complex)-mediated antigen processing and presentation. This bio-marker is related to growth factor withdrawal-induced apoptosis of myeloid precursor cells [42]. Another example is Breast Cancer Anti-Estrogen Resistance Protein 1, Crk-Associated Substrate (BCAR1). Overexpression of BCAR1 is usually detected in many cancers such as breast cancer, lung cancer, anaplastic large cell lymphoma and chronic myelogenous leukemia [43].

### 3.4. Hematopoietic Cancer Subtype Classification Model Evaluation

For evaluating the hematopoietic cancer subtype classification, we used six classification performance metrics: accuracy (*Acc*), precision (*Pre*), recall (*Rec*), the harmonic mean of precision and recall, which is called F1-measure (*F*1), geometric mean (G-mean, *GM*) and index of balanced accuracy (*IBA*, α=0.1). Furthermore, we generated a confusion matrix and a precision–recall curve (PR-curve) which were used for evaluating the imbalanced data classification performance. The equations below indicate the classification performance measurement.

(3) $$Accuracy\;\left(Acc\right)=\frac{TP+TN}{TP+TN+FP+FN}\;Precision\;\left(Pre\right)=\frac{TP}{TP+FP}\;Recall\;\left(Rec\right)=Sensitivity=\frac{TN}{TN+FN}\;Specificity\;\left(Spe\right)=\frac{TN}{TN+FP}\;F1-measure\;\left(F1\right)=\frac{2\times Rec\times Pre}{Rec+Pre}=\frac{2\times TP}{2\times TP+FP+FN}\;Geometric\;mean\;\left(GM\right)=\sqrt{Rec\times Spe}\;IBA=\left\{1+\alpha \times \left(Rec-Spe\right)\right\}\times GM$$

where *TP*, *TN*, *FP* and *FN* are the acronyms of true positive, true negative, false positive and false negative, respectively. *TP* and *TN* are the numbers of subtypes correctly classified into positive class or negative class, respectively; *FP* represents the number of instances of incorrect classification into positive class. Similarly, *FN* is the number of instances of incorrect classification into negative class.

Furthermore, for verifying the DAE-based cancer subtype classification models, we compared these models with traditional statistics and machine learning-based classification algorithms such as logistic regression (LR) [44,45], random forest (RF) [46,47], k-nearest neighbor (KNN) [48], artificial neural network (ANN) [49] and support vector machine (SVM) [50]. For the class imbalance problem on the classification task, we used an oversampling algorithm named the synthetic minority oversampling technique (SMOTE) [51,52].

**Figure 8:**
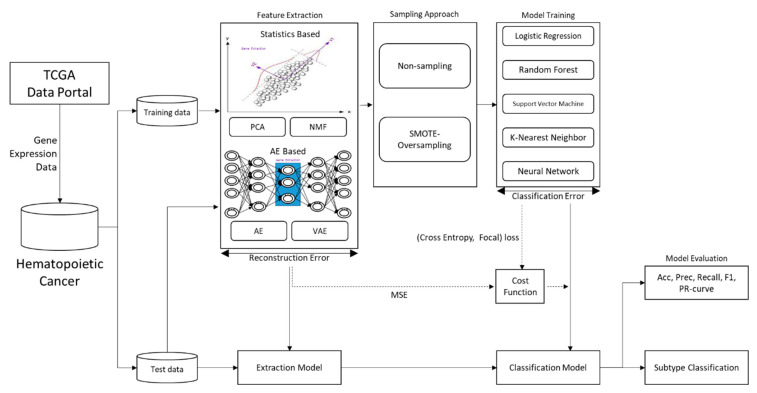
The experimental flowchart of hematopoietic cancer subtype classification.

The overall designed flowchart of this experiment is shown in Figure 8.

Table 3, Table 4, Table 5, Table 6 and Table 7 represent the results of all combinations of the experiments, which include statistical approaches (PCA, NMF) and deep learning approaches (AE, VAE). Each results table includes SMOTE for handling class imbalance on the dataset. The CE, FL, RE, TOC and TOF keywords in the loss function column represent cross-entropy, focal loss, reconstruction error, cross-entropy + reconstruction error and focal loss + reconstruction error respectively.

The results of hematopoietic cancer subtype classification using the logistic regression classification algorithm are shown in Table 3. The result of AE using merged loss, which combined cross-entropy loss and reconstruction loss, with SMOTE shows the highest results on accuracy (96.33%), recall (91.73%) and F1-measure (91.99%). In addition, the result of AE using reconstruction error with SMOTE shows the highest result on G-mean (95.33%) and IBA (95.24%).

Table 4 shows the results of hematopoietic cancer subtype classification using the k-nearest neighborhood classification algorithm. The result of AE using merged loss, which combined cross-entropy loss and reconstruction loss, with SMOTE shows the highest results on accuracy (96.06%), recall (93.82%) and F1-measure (91.31%), and the result of AE using merged loss, which combined focal loss and reconstruction error, with SMOTE shows the highest results on specificity (99.12%), G-mean (96.59%) and IBA (92.84%).

The results of hematopoietic cancer subtype classification using the random forest classification algorithm are shown in Table 5. The result of AE using merged loss, which combined with cross-entropy loss and reconstruction loss, with SMOTE shows the highest results on accuracy (96.60%), and F1-measure (91.82%), and the result of AE using reconstruction error with SMOTE shows the highest results on G-mean (99.12%), and IBA (90.80%).

In Table 6, the results of hematopoietic cancer subtype classification using the support vector machine classification algorithm are shown. The result of AE using merged loss, which combined focal loss and reconstruction loss, with SMOTE shows the best results on the evaluation matrices of accuracy (97.01%), precision (92.68%), recall (94.60%), specificity (99.52%), F1-measure (93.54%), G-mean (97.87%) and IBA (95.46%).

In Table 7, the results of hematopoietic cancer subtype classification using the artificial neural network classification algorithm are shown. The result of AE using merged loss, which combined cross-entropy loss and reconstruction loss, with SMOTE shows the highest results on accuracy (96.74%), precision (93.62%) and F1-measure (92.47%), and the result of AE using reconstruction error without the oversampling method shows the highest results on G-mean (95.39%) and IBA (90.32%).

Combining all of above results, the top 10 results based on F1-measure are summarized in Table 8. As a summary of this experiment, we found that the result of autoencoder feature selection based on the support vector machine classification algorithm with the total loss, which combined focal loss and reconstruction loss, with SMOTE showed the best performance in accuracy (97.01%), recall (94.60%), specificity (99.52%), F1-measure (93.53%), G-mean (97.87%) and IBA (95.46%).

**Figure 9:**
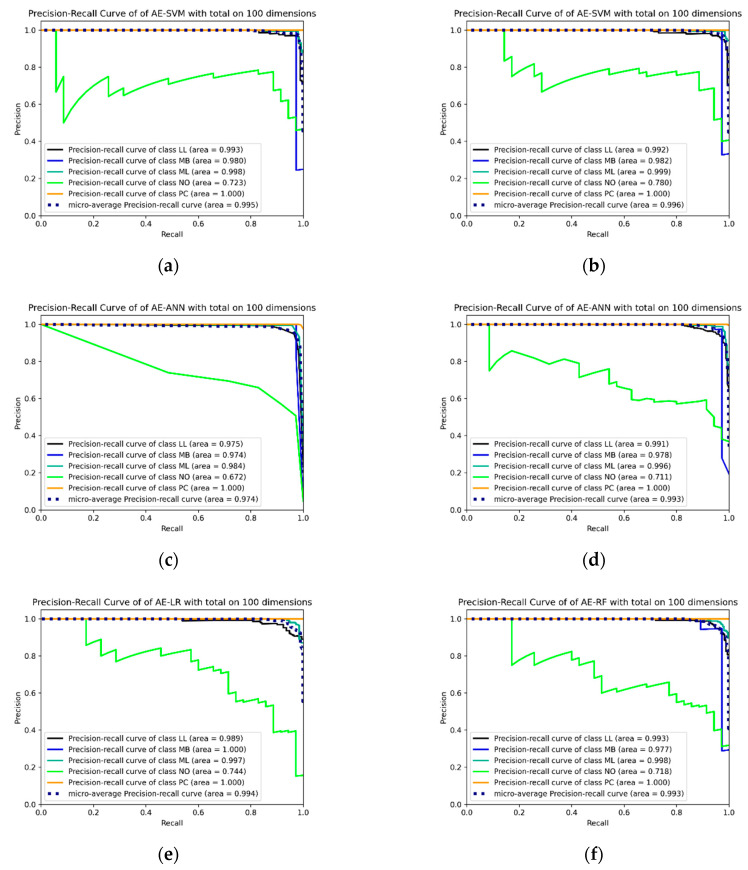
Precision–recall curves (PR-curves) for the top six hematopoietic subtype cancer classification results by F1-measure. (**a**) PR-curve for the autoencoder using FL + RE with synthetic minority oversampling technique (SMOTE) on support vector machine (SVM) classifier; (**b**) PR-curve for the autoencoder using CL + RE with SMOTE on SVM classifier; (**c**) PR-curve for the autoencoder using CL + RE with SMOTE on artificial neural network (ANN) classifier; (**d**) PR-curve for the autoencoder using FL + RE with SMOTE on ANN classifier; (**e**) PR-curve for the autoencoder using CL + RE with SMOTE on LR classifier; (**f**) PR-curve for the autoencoder using CL + RE with SMOTE on RF classifier.

**Figure 10:**
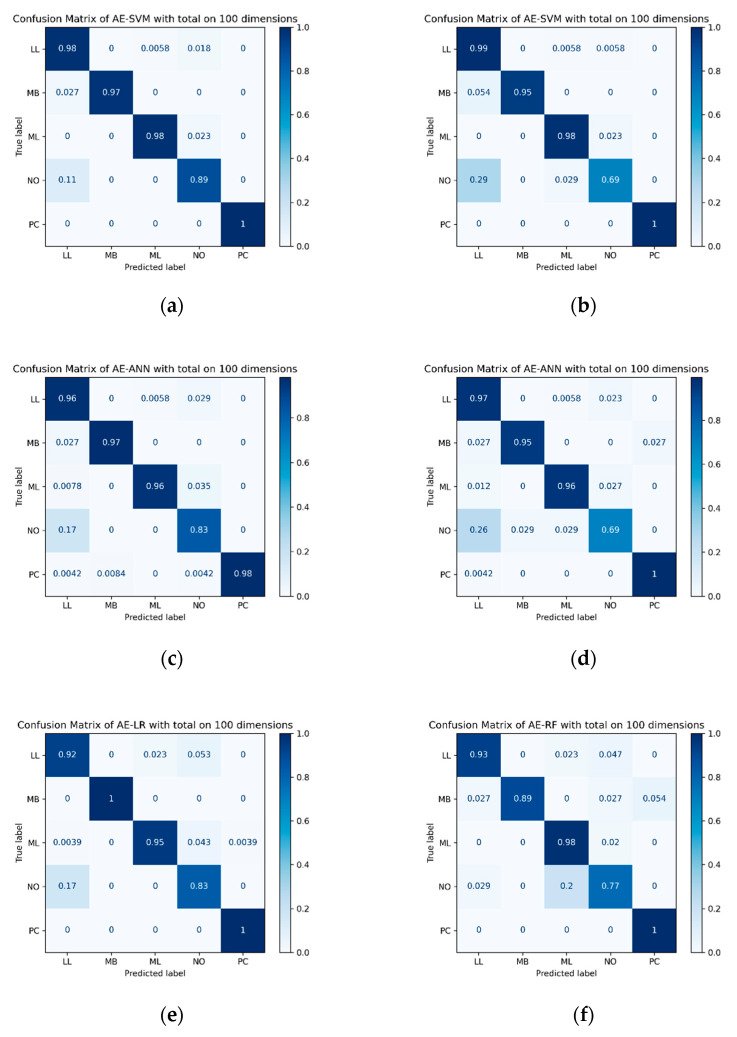
Confusion matrix for the top six hematopoietic subtype cancer classification results by F1-measure. (**a**) represents the confusion matrix for the autoencoder using FL + RE with SMOTE on SVM classifier; (**b**) represents the confusion matrix for the autoencoder using CL + RE with SMOTE on SVM classifier; (**c**) represents the confusion matrix for the autoencoder using CL + RE with SMOTE on ANN classifier; (**d**) represents the confusion matrix for the autoencoder using FL + RE with SMOTE on ANN classifier; (**e**) represents the confusion matrix for the autoencoder using CL + RE with SMOTE on LR classifier; (**f**) represents the confusion matrix for the autoencoder using CL + RE with SMOTE on RF classifier.

Figure 9, below, shows the top six PR-curves and Figure 10 shows the top six confusion matrices among the results shown in Table 8.

## 4. Discussion

In this paper, we suggested an autoencoder-based feature extraction approach for hematopoietic cancer subtype classification. The five major hematopoietic cancer subtypes were selected to create experimental data based on gene expression level. We also compared our approach with traditional feature extraction algorithms, PCA and NMF, which are widely used in cancer classification based on gene expression data. In addition, in consideration of the class imbalance problem occurring in multi-label classification, we applied the SMOTE oversampling algorithm.

In the experimental results, the traditional feature selection approaches, NMF and PCA, showed good performance, but our proposed DAE-based approach for subtype classification showed a better performance. For example, in the results of the SVM classifier using the SMOTE oversampling method, the PCA and NMF feature extraction approaches showed 90.63% and 90.22% accuracy, respectively, and the AE-based feature extraction approaches with cross-entropy error (CE), reconstruction error (RE) and merged error (CE + RE) showed 93.34%, 96.06% and 96.88% classification accuracy, respectively. This result was also the same when focal loss was applied instead of cross-entropy loss. The accuracy for each focal loss case that applied the AE-based feature extraction approach with focal loss (FL), reconstruction error (RE) and merged error (FL + RE) was 90.08%, 95.65% and 97.01%, respectively. Although SVM showed the best result with merged error (FL + RE) using focal loss, in other cases, we found that the merged error (CE + RE) using cross-entropy error showed the best performance in the other feature extraction approaches. Using those extracted results on classification algorithm, the result of subtype classification using the DAE-based feature selection approach showed better performance than traditional statistics and machine learning feature extraction approaches.

Furthermore, as shown in Table 8, when all of the results were summarized, we found that the AE-based feature extraction approach shows better performance than other feature extraction methods. In addition, when comparing the loss function, the results of applying both the classification error (FL/CE) and the reconstruction error (RE) together showed better performance rather than the single loss function, and the sampling method also showed better results when applying the SMOTE oversampling technique.

## 5. Conclusions

In this paper, we focused on the autoencoder-based feature extraction method to extract biological information from complex cancer data such as gene expression, clinical data and methylation data. We evaluated the proposed method on TCGA data samples from 2457 patients with hematopoietic cancer: lymphoid leukemia, myeloid leukemia, leukemia nos (not otherwise specified), mature B-cell leukemia and plasma cell neoplasm. To the best of our knowledge, there is no other research work on hematopoietic cancer using deep learning-based feature extraction techniques. We compared the proposed autoencoder-based method to the traditional state-of-the-art algorithms PCA and NMF, as well as another generative deep learning technique, VAE. We provided comprehensive experimental results that show the efficiency of our proposed method.

As shown in the experimental results, the proposed method shows higher performance than the other compared techniques in terms of various evaluation metrics. The proposed method with TOF loss achieved the highest accuracy (97.01%), precision (92.68%), recall (94.60%), specificity (99.52%), F1-measure (93.53%), G-mean (97.87%) and index imbalanced accuracy (95.46%) followed by the SVM classifier, which was trained on the sampled data by SMOTE. The learned representations contain rich, valuable information related to hematopoietic cancer which also can be used for other downstream tasks such as regression, classification, survival analysis, etc. We also applied the SHAP feature interpretation technique to our pre-trained model to explain the black box and show the importance of each bio-marker. By extracting bio-markers using deep learning structures, this study is expected to be helpful in enabling gene-specific treatment of patients. Furthermore, it is expected that this model will be helpful in the development of public healthcare through extensibility that can be applied not only to cancer but also to various diseases.

In conclusion, we found that our autoencoder-based feature extraction approach for hematopoietic cancer subtype classification algorithm showed good classification performance in multiclass classification, and our suggested approach showed better performance than PCA and NMF, which are widely used feature extraction methods for cancer classification. Furthermore, the problem of unbalanced data can be solved by applying the SMOTE method.

## Figures and Tables

**Table 1:** Summary of the hematopoietic cancer dataset.

Subtype	# Sample	Train	Test
Lymphoid leukemias (LL)	550	440	110
Myeloid leukemias (ML)	818	654	164
Leukemia nos (NO)	104	83	21
Mature B-cell leukemias (MB)	113	90	23
Plasma cell neoplasms (PC)	860	688	172

**Table 2:** Top 20 gene/protein bio-markers produced by AE and Shapley Additive exPlanations (SHAP).

No	Code	Gene/Protein
1	ENSG00000113269.12	RNF130
2	ENSG00000050820.15	BCAR1
3	ENSG00000180535.3	BHLHA15
4	ENSG00000167861.14	HID1
5	ENSG00000126264.8	HCST
6	ENSG00000183508.4	TENT5C
7	ENSG00000173715.14	C11orf80
8	ENSG00000149054.13	ZNF215
9	ENSG00000104635.12	SLC39A14
10	ENSG00000182511.10	FES
11	ENSG00000134285.9	FKBP11
12	ENSG00000171067.9	C11orf24
13	ENSG00000258227.5	CLEC5A
14	ENSG00000258013.2	RPL3P13
15	ENSG00000076554.14	TPD52
16	ENSG00000110665.10	C11orf21
17	ENSG00000110777.10	POU2AF1
18	ENSG00000197879.13	MYO1C
19	ENSG00000177045.7	SIX5
20	ENSG00000151729.9	SLC25A4

**Table 3:** Evaluation matrices for subtype classification using logistic regression (LR).

Feature Extraction	Loss	Sampling	Acc	Pre	Rec	Spe	F1	GM	IBA
PCA	No	No	0.9348	0.8843	0.8733	0.9833	0.8772	0.9267	0.8492
		SMOTE	0.9361	0.8874	0.8893	0.9867	0.8852	0.9493	0.8946
NMF	No	No	0.9416	0.8729	0.8453	0.9850	0.8515	0.9125	0.8210
		SMOTE	0.9416	0.8761	0.9008	0.9854	0.8876	0.9417	0.8792
AE	CE	No	0.6563	0.3869	0.3972	0.8541	0.3398	0.5124	0.2482
		SMOTE	0.8207	0.7799	0.8358	0.9317	0.7376	0.8552	0.7207
	RE	No	0.9457	**0.9284**	0.8388	0.9890	0.8504	0.9319	0.8589
		SMOTE	0.9524	0.8916	0.9054	0.9888	0.8970	**0.9533**	**0.9024**
	TOC	No	0.9552	0.9065	0.8738	0.9861	0.8858	0.9215	0.8385
		SMOTE	**0.9633**	0.9229	**0.9173**	0.9882	**0.9199**	0.9515	0.8988
	FL	No	0.4878	0.3914	0.2984	0.8518	0.2484	0.5097	0.2456
		SMOTE	0.6808	0.6361	0.7263	0.9435	0.6049	0.8580	0.7242
	RE	No	0.9348	0.8946	0.8248	0.9787	0.8352	0.9176	0.8320
		SMOTE	0.9429	0.8861	0.8686	0.9770	0.8747	0.9272	0.8513
	TOF	No	0.9633	0.9103	0.9079	0.9886	0.9088	0.9304	0.8559
		SMOTE	0.9592	0.8991	0.9279	**0.9899**	0.9114	0.9516	0.8987
VAE	CE	No	0.3125	0.1814	0.1979	0.7998	0.1852	0.3997	0.1502
		SMOTE	0.1984	0.1954	0.1560	0.7974	0.1618	0.3951	0.1467
	RE	No	0.3234	0.2118	0.2078	0.7962	0.1968	0.3836	0.1382
		SMOTE	0.2215	0.2067	0.2149	0.7998	0.1893	0.4016	0.1517
	TOC	No	0.3505	0.2649	0.2299	0.7923	0.2233	0.3956	0.1472
		SMOTE	0.2418	0.2413	0.2317	0.7985	0.2092	0.4105	0.1586
	FL	No	0.3111	0.2120	0.2639	0.8130	0.1961	0.4360	0.1791
		SMOTE	0.2106	0.1971	0.1876	0.8000	0.1752	0.3816	0.1366
	RE	No	0.3288	0.2230	0.2110	0.8021	0.2039	0.4026	0.1524
		SMOTE	0.2174	0.2174	0.2209	0.7941	0.1924	0.3759	0.1326
	TOF	No	0.3152	0.1916	0.2006	0.8004	0.1884	0.4003	0.1506
		SMOTE	0.2269	0.2096	0.2082	0.8018	0.1894	0.3930	0.1451

CE = Cross entropy loss, FL = Focal loss, RE = Reconstruction error, TOC = CE + RE, TOF = FL + RE, Bold = The highest score.

**Table 4:** Evaluation matrices for subtype classification using k-nearest neighbor (KNN).

Feature Extraction	Loss	Sampling	Acc	Pre	Rec	Spe	F1	GM	IBA
PCA	No	No	0.9171	0.8293	0.8047	0.9787	0.8136	0.8874	0.7738
		SMOTE	0.9171	0.8225	0.8317	0.9794	0.8248	0.9025	0.8025
NMF	No	No	0.9117	0.8127	0.8049	0.9772	0.8040	0.8869	0.7730
		SMOTE	0.9185	0.8294	0.8602	0.9798	0.8428	0.9180	0.8327
AE	CE	No	0.9389	0.8888	0.8235	0.9870	0.8341	0.9195	0.8345
		SMOTE	0.9402	0.8685	0.9074	0.9850	0.8830	0.9525	0.9015
	RE	No	0.9307	0.8908	0.8242	0.9866	0.8366	0.9155	0.8267
		SMOTE	0.9117	0.8123	0.8651	0.9842	0.8341	0.9376	0.8712
	TOC	No	0.9538	**0.9306**	0.8637	0.9873	0.8788	0.9144	0.8243
		SMOTE	**0.9606**	0.8934	**0.9382**	0.9860	**0.9131**	0.9401	0.8760
	FL	No	0.9198	0.8576	0.7990	0.9783	0.7879	0.8711	0.7435
		SMOTE	0.8927	0.8060	0.8605	0.9777	0.8209	0.9107	0.8187
	RE	No	0.9402	0.8875	0.8441	0.9802	0.8494	0.8971	0.7920
		SMOTE	0.9185	0.8059	0.8647	0.9770	0.8290	0.9242	0.8453
	TOF	No	0.9524	0.8937	0.8758	0.9900	0.8773	0.9270	0.8488
		SMOTE	0.9457	0.8554	0.9365	**0.9912**	0.8855	**0.9659**	**0.9284**
VAE	CE	No	0.3207	0.2094	0.2108	0.7959	0.2056	0.3864	0.1402
		SMOTE	0.0918	0.3292	0.1843	0.7991	0.0909	0.3946	0.1463
	RE	No	0.3356	0.3459	0.2265	0.7971	0.2258	0.3884	0.1417
		SMOTE	0.1087	0.1345	0.2258	0.7966	0.0997	0.3701	0.1284
	TOC	No	0.3166	0.2615	0.2166	0.7996	0.2157	0.3965	0.1478
		SMOTE	0.1168	0.1397	0.2424	0.8011	0.1059	0.4075	0.1562
	FL	No	0.2826	0.1707	0.1900	0.9787	0.1760	0.8874	0.7738
		SMOTE	0.0951	0.1475	0.1797	0.9794	0.0855	0.9025	0.8025
	RE	No	0.3057	0.1852	0.2011	0.9772	0.1923	0.8869	0.7730
		SMOTE	0.0883	0.1473	0.1710	0.9798	0.0813	0.9180	0.8327
	TOF	No	0.2988	0.1691	0.1850	0.9870	0.1762	0.9195	0.8345
		SMOTE	0.1101	0.2022	0.2265	0.9850	0.1012	0.9525	0.9015

CE = Cross entropy loss, FL = Focal loss, RE = Reconstruction error, TOC = CE + RE, TOF = FL + RE, Bold = The highest score.

**Table 5:** Evaluation matrices for subtype classification using random forest (RF).

Feature Extraction	Loss	Sampling	Acc	Pre	Rec	Spe	F1	GM	IBA
PCA	No	No	0.9416	0.8836	0.8444	0.9869	0.8584	0.9232	0.8417
		SMOTE	0.9511	0.8994	0.8886	0.9878	0.8936	0.9369	0.8691
NMF	No	No	0.9402	0.9053	0.8247	0.9845	0.8381	0.9019	0.8005
		SMOTE	0.9511	0.9165	0.8802	0.9875	0.8951	0.9351	0.8655
AE	CE	No	0.9212	**0.9470**	0.7616	0.9660	0.7668	0.8231	0.6595
		SMOTE	0.9389	0.8857	0.8439	0.9686	0.8620	0.8637	0.7313
	RE	No	0.9429	0.9118	0.8091	0.9874	0.8228	0.9081	0.8120
		SMOTE	0.9592	0.9114	0.9107	0.9901	0.9109	**0.9561**	**0.9080**
	TOC	No	0.9511	0.9307	0.8335	0.9867	0.8519	0.9060	0.8082
		SMOTE	**0.9660**	0.9289	0.9102	0.9888	**0.9182**	0.9391	0.8734
	FL	No	0.8682	0.7197	0.7423	0.9759	0.7257	0.8610	0.7253
		SMOTE	0.8886	0.8569	0.7990	0.9774	0.8055	0.8874	0.7739
	RE	No	0.9497	0.9359	0.8272	0.9780	0.8384	0.8803	0.7605
		SMOTE	0.9620	0.9123	0.9021	0.9853	0.9067	0.9336	0.8628
	TOF	No	0.9470	0.9044	0.8393	0.9882	0.8513	0.9144	0.8242
		SMOTE	0.9606	0.9131	**0.9157**	**0.9911**	0.9142	0.9487	0.8926
VAE	CE	No	0.3696	0.2268	0.2258	0.7936	0.1938	0.3864	0.1403
		SMOTE	0.3016	0.1677	0.1879	0.7974	0.1762	0.3927	0.1449
	RE	No	0.3261	0.1764	0.1972	0.8023	0.1618	0.4063	0.1552
		SMOTE	0.3166	0.1824	0.2017	0.7969	0.1905	0.3907	0.1434
	TOC	No	0.3682	0.1822	0.2229	0.7943	0.1822	0.3868	0.1405
		SMOTE	0.2894	0.2216	0.1913	0.7978	0.1877	0.3953	0.1468
	FL	No	0.3465	0.2101	0.2104	0.8049	0.1765	0.4108	0.1587
		SMOTE	0.2840	0.2072	0.1895	0.7989	0.1844	0.3963	0.1476
	RE	No	0.3111	0.1735	0.1897	0.8067	0.1588	0.4146	0.1617
		SMOTE	0.3207	0.3877	0.2109	0.8031	0.2054	0.4055	0.1546
	TOF	No	0.3329	0.1798	0.2026	0.8004	0.1700	0.4012	0.1513
		SMOTE	0.2745	0.2227	0.1777	0.8077	0.1727	0.4171	0.1637

CE = Cross entropy loss, FL = Focal loss, RE = Reconstruction error, TOC = CE + RE, TOF = FL + RE, Bold = The highest score.

**Table 6:** Evaluation matrices for subtype classification using support vector machine (SVM).

Feature Extraction	Loss	Sampling	Acc	Pre	Rec	Spe	F1	GM	IBA
PCA	No	No	0.8614	0.8709	0.6119	0.9613	0.6202	0.7670	0.5677
		SMOTE	0.9063	0.7963	0.8620	0.9778	0.816	0.9181	0.8332
NMF	No	No	0.8696	0.7725	0.6141	0.9627	0.6215	0.7689	0.5705
		SMOTE	0.9022	0.7806	0.8453	0.9778	0.7994	0.9104	0.8181
AE	CE	No	0.9253	0.7492	0.7722	0.9746	0.7589	0.8645	0.7318
		SMOTE	0.9334	0.8690	0.8902	0.9773	0.8764	0.9245	0.8459
	RE	No	0.9457	0.8994	0.8349	0.9887	0.8527	0.9378	0.8708
		SMOTE	0.9606	0.9158	0.9164	0.9897	0.9160	0.9647	0.9261
	TOC	No	0.9579	0.9173	0.8759	0.9894	0.8886	0.9332	0.8614
		SMOTE	0.9688	0.9256	0.9309	0.9911	0.9281	0.9601	0.9162
	FL	No	0.9171	0.7445	0.7720	0.9767	0.7562	0.8663	0.7349
		SMOTE	0.9008	0.8353	0.8633	0.9787	0.8404	0.9177	0.8322
	RE	No	0.9538	0.9121	0.8485	0.9814	0.8630	0.9021	0.8013
		SMOTE	0.9565	0.8981	0.8936	0.9846	0.8952	0.9371	0.8700
	TOF	No	0.9592	0.9116	0.8865	0.9929	0.8968	0.9554	0.9061
		SMOTE	**0.9701**	**0.9268**	**0.9460**	**0.9952**	**0.9354**	**0.9787**	**0.9546**
VAE	CE	No	0.3261	0.1674	0.1981	0.7941	0.1600	0.3867	0.1405
		SMOTE	0.3003	0.1672	0.1874	0.7951	0.1701	0.3898	0.1428
	RE	No	0.3234	0.1316	0.1944	0.7983	0.1550	0.3962	0.1475
		SMOTE	0.3165	0.1877	0.1967	0.7967	0.1761	0.3916	0.1441
	TOC	No	0.3872	0.2027	0.2352	0.7961	0.1926	0.3907	0.1434
		SMOTE	0.375	0.2080	0.2328	0.7995	0.2096	0.3982	0.1490
	FL	No	0.3179	0.2273	0.1931	0.8088	0.1553	0.4192	0.1654
		SMOTE	0.3098	0.1740	0.1940	0.8135	0.1744	0.4302	0.1743
	RE	No	0.3438	0.3387	0.2075	0.8022	0.1650	0.4046	0.1539
		SMOTE	0.3220	0.1881	0.2015	0.8047	0.1824	0.4117	0.1595
	TOF	No	0.3438	0.2372	0.2080	0.8049	0.1690	0.4112	0.1590
		SMOTE	0.3356	0.1971	0.2090	0.8064	0.1904	0.4149	0.1619

CE = Cross entropy loss, FL = Focal loss, RE = Reconstruction error, TOC = CE + RE, TOF = FL + RE, Bold = The highest score.

**Table 7:** Evaluation matrices for subtype classification using artificial neural network (ANN).

Feature Extraction	Loss	Sampling	Acc	Pre	Rec	Spe	F1	GM	IBA
PCA	No	No	0.9416	0.8677	0.8431	0.9820	0.8495	0.9179	0.8320
		SMOTE	0.9280	0.8660	0.8428	0.9665	0.8434	0.8799	0.7615
NMF	No	No	0.9524	0.9028	0.8668	0.9867	0.8783	0.9225	0.8404
		SMOTE	0.9443	0.8728	0.8899	0.9851	0.8807	0.9344	0.8645
AE	CE	No	0.9130	0.7423	0.7141	0.9677	0.7184	0.8421	0.6926
		SMOTE	0.9402	0.8798	0.9086	0.9682	0.8888	0.9173	0.8331
	RE	No	0.9592	0.9367	0.8865	0.9914	0.9028	**0.9539**	**0.9032**
		SMOTE	0.9592	0.9318	0.8910	0.9893	0.9054	0.9472	0.8898
	TOC	No	0.9620	0.9250	0.8741	0.9896	0.8884	0.9386	0.8721
		SMOTE	**0.** **9674**	**0.** **9362**	0.9151	0.9880	**0.9247**	0.9353	0.8658
	FL	No	0.9022	0.7005	0.7589	0.9675	0.7275	0.8267	0.6656
		SMOTE	0.8777	0.8047	0.8490	0.9763	0.8124	0.9126	0.8225
	RE	No	0.9620	0.9180	0.9058	0.9846	0.9111	0.9284	0.8525
		SMOTE	0.9565	0.9086	0.8970	0.9826	0.9015	0.9243	0.8448
	TOF	No	0.9633	0.9235	0.8987	**0.** **9923**	0.9093	0.9515	0.8981
		SMOTE	0.9633	0.9165	**0.9268**	0.9906	0.9210	0.9385	0.8719
VAE	CE	No	0.3111	0.1986	0.2063	0.7926	0.2003	0.3754	0.1322
		SMOTE	0.2731	0.1914	0.1920	0.7976	0.1909	0.3940	0.1459
	RE	No	0.3030	0.1824	0.1944	0.7965	0.1874	0.3853	0.1394
		SMOTE	0.3030	0.2065	0.2031	0.7969	0.2006	0.3967	0.1479
	TOC	No	0.3030	0.2098	0.2005	0.8054	0.1979	0.4072	0.1559
		SMOTE	0.3315	0.2319	0.2455	0.8071	0.2473	0.4174	0.1639
	FL	No	0.2921	0.1910	0.1939	0.8068	0.1893	0.4162	0.1630
		SMOTE	0.3152	0.2256	0.2210	0.8001	0.2208	0.3988	0.1495
	RE	No	0.2677	0.1737	0.1772	0.8029	0.1732	0.4062	0.1551
		SMOTE	0.3043	0.2220	0.2213	0.8031	0.2205	0.4145	0.1617
	TOF	No	0.3193	0.2127	0.2091	0.8064	0.2052	0.4114	0.1592
		SMOTE	0.2935	0.1934	0.1956	0.8005	0.1929	0.4203	0.1664

CE = Cross entropy loss, FL = Focal loss, RE = Reconstruction error, TOC = CE + RE, TOF = FL + RE, Bold = The highest score.

**Table 8:** Top 10 performances for hematopoietic cancer subtype classification ordered by F1-measure.

	Feature Extraction	Loss	Sampling	Acc	Pre	Rec	Spe	F1	GM	IBA
SVM	AE	TOF	SMOTE	**0.9701**	0.9268	**0.9460**	**0.** **9952**	**0.9354**	**0.9787**	**0.9546**
SVM	AE	TOC	SMOTE	0.9688	0.9256	0.9309	0.9911	0.9281	0.9601	0.9162
ANN	AE	TOC	SMOTE	0.9674	**0.9362**	0.9151	0.988	0.9247	0.9353	0.8658
ANN	AE	TOF	SMOTE	0.9633	0.9165	0.9268	0.9906	0.921	0.9385	0.8719
LR	AE	TOC	SMOTE	0.9633	0.9229	0.9173	0.9882	0.9199	0.9515	0.8988
RF	AE	TOC	SMOTE	0.966	0.9289	0.9102	0.9888	0.9182	0.9391	0.8734
SVM	AE	RE	SMOTE	0.9606	0.9158	0.9164	0.9897	0.916	0.9647	0.9261
RF	AE	TOF	SMOTE	0.9606	0.9131	0.9157	0.9911	0.9142	0.9487	0.8926
KNN	AE	TOC	SMOTE	0.9606	0.8934	0.9382	0.986	0.9131	0.9401	0.876
LR	AE	TOF	SMOTE	0.9592	0.8991	0.9279	0.9899	0.9114	0.9516	0.8987

CE = Cross entropy loss, FL = Focal loss, RE = Reconstruction error, TOC = CE + RE, TOF = FL + RE, Bold = The highest score.

**Figure A1:**
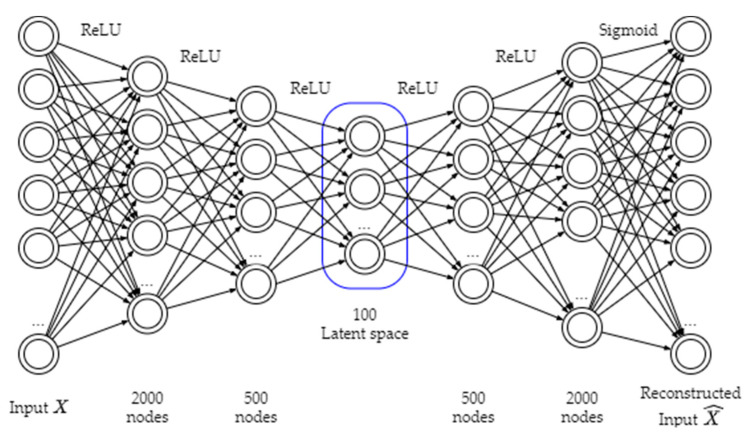
The procedure of autoencoder (AE) feature selection method. The encoder consists of three fully connected hidden layers which contain 2000, 500 and 100 nodes and the decoder consists of two fully connected hidden layers which contains 500 and 2000 nodes. This autoencoder structure is evaluated by reconstruction error using MSE measurement.

**Figure A2:**
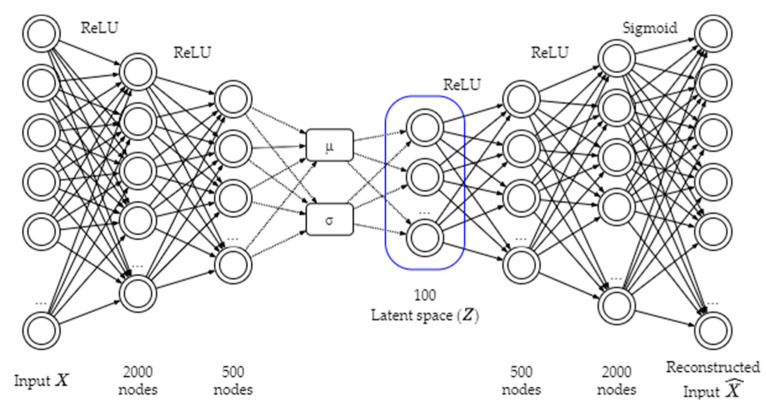
The procedure of the variational autoencoder (VAE) feature selection method. The encoder consists of three fully connected hidden layers which contain 2000, 500 and 100 nodes and the decoder consists two fully connected hidden layers which contain 500 and 2000 nodes. Unlike the autoencoder, this method uses Kullback–Leibler divergence (KL-divergence) for calculating reconstruction loss for variational autoencoder.

## Appendix A. Autoencoder

In this paper, we used an autoencoder (AE) and a variational autoencoder (VAE) as non-linear feature extraction methods. The structure of an AE is divided into two main parts: encoder and decoder. The encoder has an input layer with three fully connected hidden layers with 2000, 500 and 100 nodes. The decoder is comprised of two hidden layers, which are fully connected. These layers transpose the weights of the encoding and decoding using the equations below:

(A1) $$hidden_{encode_{1}}=ReLU\left(W_{1}\times input+b_{1}\right)\;hidden_{encode_{2}}=ReLU\left(W_{2}\times hidden_{encode_{1}}+b_{2}\right)\;hidden_{encode_{3}}=ReLU\left(W_{3}\times hidden_{encode_{2}}+b_{3}\right)\;hidden_{decode_{1}}=ReLU\left(W_{3}{}^{’}\times hidden_{encode_{3}}+b_{3}{}^{’}\right)\;hidden_{decode_{2}}=ReLU\left(W_{2}{}^{’}\times hidden_{decode_{1}}+b_{2}{}^{’}\right)\;reconstruction_{input}=sigmoid\left(W_{1}{}^{’}\times hidden_{decode_{2}}+b_{1}{}^{’}\right)$$

where, $W_{1}$, $W_{2}$ and $W_{3}$ are the weight vectors between each layer. Assume that the input size is N, the size of each $W_{1}$, $W_{2}$ and $W_{3}$ is N×2000, 2000×500 and 500×100, respectively. The $b_{1}$, $b_{2}$ and $b_{3}$ are the bias information for each layer. Rectified Linear Unit (ReLU) and sigmoid function were applied for non-linear activation functions. The $hidden_{decode_{1}}$ was used as the extracted features of AE. For measuring reconstruction loss, we used MSE as a loss function between the original data ($x_{i}$) and reconstructed data (${\hat{x}}_{i}$). The equation of MSE is shown below.

(A2) $$MSE=\frac{1}{N}\sum^{\;}{\left(x_{i}-{\hat{x}}_{i}\right)}^{2}$$

This sequence of autoencoder processes is shown in Figure A1.

## Appendix B. Variational Autoencoder

Similar to the AE structure, the VAE also has an encoder and a decoder. This VAE is normally used in semi-supervised learning models nowadays. Furthermore, it can learn approximate inferences and be trained using the gradient descent method. The main approach of VAEs is using probability distribution to obtain samples which match the distribution. This VAE calculates objective function using reconstruction loss and Kullback–Leibler divergence (KLdivergence) [38].

(A3) $$ℒ\left(X\right)=E\left[\log P\left(X|Z\right)\right]-D_{KL}\left[Q\left(Z|X\right)||P\left(Z\right)\right]$$

where ℒ is a VAE objective function, which is known as a variational lower bound; X is input data and Z is a latent variable. The left term of VAE is the reconstruction loss—it makes the decoder reconstruct input. The right term is referred to as KL-divergence for minimizing the difference between the encoder’s distribution Q(Z|X) and prior distribution P(Z). For calculating distribution, it generates mean and standard deviation. The goal of this objective function is to maximize the variational lower bound using the maximization of data generation and minimization the KL-divergence. In this paper, the VAE shared the construction with AE; however, due to the VAE considering the distribution of the input, the VAE applies the distribution calculating part before generating the latent space. Almost the same as the AE, the VAE transposes the weights of the encoding and decoding using the below equations:

(A4) $$hidden_{encode_{1}}=ReLU\left(W_{1}\times input+b_{1}\right)\;hidden_{encode_{2}}=ReLU\left(W_{2}\times hidden_{encode_{1}}+b_{2}\right)\;hidden_{encode_{3}}=E\left[\log P\left(X|Z\right)\right]-D_{KL}\left[Q\left(Z|X\right)||P\left(Z\right)\right]\;hidden_{decode_{1}}=ReLU\left(W_{3}\times hidden_{encode_{3}}+b_{3}\right)\;hidden_{decode_{2}}=ReLU\left(W_{2}{}^{’}\times hidden_{decode_{1}}+b_{2}{}^{’}\right)\;reconstruction_{input}=sigmoid\left(W_{1}{}^{’}\times hidden_{decode_{2}}+b_{1}{}^{’}\right)$$

where, $W_{1}$, $W_{2}$ and $W_{3}$ are the weight vectors between each layer. Assume that the input size is N, the size of $W_{1}$ and $W_{2}$ is N×2000 and 2000×500 respectively. In the third layer on the encoder, the distribution of input is computed and the mean (μ) and standard deviation (σ) of input are generated for calculating latent space (Z). The $b_{1}$, $b_{2}$ and $b_{3}$ are the bias information for each layer. In VAE, ReLU and sigmoid functions are also used as non-linear activation functions. The result $hidden_{decode_{1}}$, called latent space (Z), is used as the extracted feature of the VAE. For measuring reconstruction loss, MSE is used as a loss function between the original data and reconstructed data, same as the AE.

This sequence of variational autoencoder processes is shown in Figure A2.

## Appendix C. Subtype Classification Neural Network

For the NN for the subtype classification on hematopoietic cancer, we generated a feed-forward neural network (NN), which has one input layer, one hidden layer with 100 nodes and one output layer. The features we extracted from DAE utilized the input of NN.

(A5) $$hidden_{layer}=ReLU\left(W_{4}\times hidden_{encode_{3}}+b_{4}\right)\;output=sigmoid\left(W_{5}\times hidden_{layer}+b_{5}\right)$$

where $W_{4}$ and $W_{5}$ indicate the weight vector between the layers between 100×100 and 100×C; C indicates the number of class labels—in this case, it is 5—and $b_{4}$ and $b_{5}$ are the bias of each NN layer’s node.

## Appendix D. Cross-Entropy Loss and Focal Loss

For calculating classification loss, we used two loss functions: cross-entropy loss (CE) and focal loss (FL). CE is the most widely used loss function in classification models. The equation of CE is shown in the below equation:

(A6) $$CE\left(p,y\right)\;=-\overset{N}{\underset{i=1}{\sum }}y_{i}\log\left(P\left(i|S\right)\right)$$

where y is the class label of the instance; N is the number of classification labels on the dataset; P(i|S) is the predicted probability. If the model predicts an instance correctly as $y_{i}$, P(i|S) heads to the true distribution of $y_{i}$. As a result, the loss function will be decreased. For simplicity, if $p_{i}$ is defined in the estimated probability of the model when class label $y_{i}$ = 1, it can be written by the below equation:

(A7) $$CE\left(p,y\right)=CE\left(p_{i}\right)=-\log\left(p_{i}\right)$$

However, if there are plenty of class labels with the imbalance status, it incurs a loss with the non-trivial magnitude. If there are a lot of easy examples to be classified, which means there is a large class imbalance on the dataset, the CE of major class labels is going to be larger than the minor classes.

For handling this class imbalance problem, a modulating factor is added to CE, defined as focal loss (FL). This modulating factor in focal loss is ${\left(1-p_{i}\right)}^{\gamma }$. Here, γ is a focusing factor which can be changeable parameter that ranges γ≥0. Therefore, the FL can be formulated as the below equation:

(A8) $$FL\left(p,y\right)=FL\left(p_{i}\right)=-{\left(1-p_{i}\right)}^{\gamma }\log\left(p_{i}\right)$$

For instance, if the focusing factor is set as γ=2 and a sample probability $p_{t}=0.9$, the loss contribution is 100 times lower than CE. On the other hand, the modulating factor makes the loss contribution of the example lower and makes it easier to classify and compare than when using traditional CE. Due to this advantage, using FL can prevent the overfitting problem which can be accompanied by class imbalance.
